# 2D cine DENSE with low encoding frequencies accurately quantifies cardiac mechanics with improved image characteristics

**DOI:** 10.1186/s12968-015-0196-z

**Published:** 2015-11-04

**Authors:** Gregory J. Wehner, Jonathan D. Grabau, Jonathan D. Suever, Christopher M. Haggerty, Linyuan Jing, David K. Powell, Sean M. Hamlet, Moriel H. Vandsburger, Xiaodong Zhong, Brandon K. Fornwalt

**Affiliations:** Department of Biomedical Engineering, University of Kentucky, Lexington, KY USA; Department of Pediatrics, University of Kentucky, Lexington, KY USA; Department of Electrical Engineering, University of Kentucky, Lexington, KY USA; MR R&D Collaborations, Siemens Healthcare, Atlanta, GA USA; Department of Physiology, University of Kentucky, Lexington, KY USA; Department of Medicine, University of Kentucky, Lexington, KY USA; Institute for Advanced Application, Geisinger Health System, Danville, PA USA; Institute for Advanced Application, Geisinger Clinic, 100 North Academy Avenue, Danville, PA 17822-4400 USA

**Keywords:** DENSE, Displacement, Cardiac mechanics, Encoding frequency, Strain, Twist, Magnetic resonance

## Abstract

**Background:**

Displacement Encoding with Stimulated Echoes (DENSE) encodes displacement into the phase of the magnetic resonance signal. The encoding frequency (k_e_) maps the measured phase to tissue displacement while the strength of the encoding gradients affects image quality. 2D cine DENSE studies have used a k_e_ of 0.10 cycles/mm, which is high enough to remove an artifact-generating echo from k-space, provide high sensitivity to tissue displacements, and dephase the blood pool. However, through-plane dephasing can remove the unwanted echo and dephase the blood pool without relying on high k_e_. Additionally, the high sensitivity comes with the costs of increased phase wrapping and intra-voxel dephasing. We hypothesized that k_e_ below 0.10 cycles/mm can be used to improve image characteristics and provide accurate measures of cardiac mechanics.

**Methods:**

Spiral cine DENSE images were obtained for 10 healthy subjects and 10 patients with a history of heart disease on a 3 T Siemens Trio. A mid-ventricular short-axis image was acquired with different k_e_: 0.02, 0.04, 0.06, 0.08, and 0.10 cycles/mm. Peak twist, circumferential strain, and radial strain were compared between acquisitions employing different k_e_ using Bland-Altman analyses and coefficients of variation. The percentage of wrapped pixels in the phase images at end-systole was calculated for each k_e_. The dephasing of the blood signal and signal to noise ratio (SNR) were also calculated and compared.

**Results:**

Negligible differences were seen in strains and twist for all k_e_ between 0.04 and 0.10 cycles/mm. These differences were of the same magnitude as inter-test differences. Specifically, the acquisitions with 0.04 cycles/mm accurately quantified cardiac mechanics and had zero phase wrapping. Compared to 0.10 cycles/mm, the acquisitions with 0.04 cycles/mm had 9 % greater SNR and negligible differences in blood pool dephasing.

**Conclusions:**

For 2D cine DENSE with through-plane dephasing, the encoding frequency can be lowered to 0.04 cycles/mm without compromising the quantification of twist or strain. The amount of wrapping can be reduced with this lower value to greatly simplify the input to unwrapping algorithms. The strain and twist results from studies using different encoding frequencies can be directly compared.

## Background

Displacement Encoding with Stimulated Echoes (DENSE) is a cardiovascular magnetic resonance (CMR) technique that encodes tissue displacement into the phase of the MR signal [1]. The resulting pixel-level resolution of the displacement field has been used to quantify cardiac mechanics in both healthy and diseased animals and humans [1–6]. The encoding gradient strength is proportional to the displacement sensitivity of the phase images. It is often referred to as the encoding frequency (k_e_) with units of cycles/mm.

**Fig. 1:**
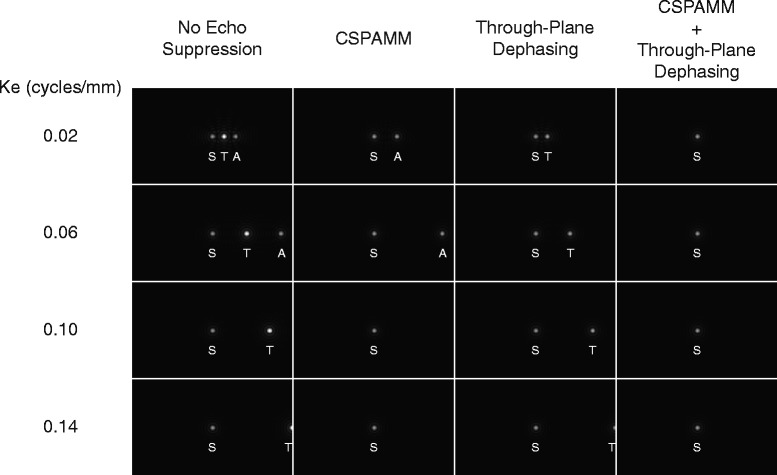
The effect of encoding frequency (k_e_) and artifact suppression techniques on the DENSE k-space. These simulations of the DENSE k-space illustrate the effect of k_e_ and artifact suppression techniques. Consider the first k-space in column 1. The echo at the center of k-space is the desired stimulated echo (S). The echo to its right is the T1 relaxation echo (T). The third echo is the stimulated anti-echo (A). Stripe artifacts are generated by the T1 echo and the anti-echo. With no echo suppression technique, a high k_e_ must be used to shift both artifact-generating echoes beyond the sampled region of k-space (column 1). With CSPAMM echo suppression, the T1 echo is suppressed (column 2). Through-plane dephasing selectively dephases the anti-echo and the T1 echo (column 3). The use of CSPAMM and through-plane dephasing together suppresses both artifact-generating echoes, which removes the dependence on high k_e_ for artifact suppression (column 4)

In addition to specifying sensitivity, the k_e_ plays a role in several other processes related to image quality and post-processing. The earliest implementations of DENSE relied on a high k_e_ to shift the artifact-generating echoes beyond the sampled region of k-space [1] (Fig. 1, column 1). While this technique removed stripe artifacts, the high encoding gradients caused significant intra-voxel dephasing in deforming tissue, which limited the ability to properly encode displacement during systole [1]. The incorporation of complementary spatial modulation of magnetization (CSPAMM) for echo suppression removed the first artifact-generating echo (the T1 relaxation echo) [3] (Fig. 1, column 2). This allowed for lower k_e_, and thus lower gradients leading to less intra-voxel dephasing, since only the furthest echo (the stimulated anti-echo) had to be shifted out of the k-space field of view. Finally, the addition of a thru-plane dephasing gradient selectively dephased the stimulated anti-echo while preserving the desired stimulated echo [7] (Fig. 1, column 4). This final addition removed the dependence on high k_e_ for artifact suppression.

A low k_e_ is desired to improve the signal to noise ratio (SNR) by reducing the amount of intra-voxel dephasing and to prevent excessive wrapping in the phase images. Recent studies with 2D DENSE have used an in-plane k_e_ of 0.10 cycles/mm, which creates wrapping in most subjects as only 5 mm of displacement is required before wrapping occurs [5, 8, 9]. Unwrapping algorithms have been developed and utilized, but they are not guaranteed to be error-free in all subjects or all regions of a given subject’s heart [10]. Regions with high velocities and noise are the most challenging for automated and semi-automated techniques. Importantly, DENSE studies that use the balanced encoding strategy and online image reconstruction suffer from up to three-fold increased phase wrapping [11] that may not be correctly resolved by the unwrapping algorithm, particularly in the presence of noise. Indeed, lower k_e_ (0.06 cycles/mm) have been used in these studies to reduce the amount of wrapping and simplify the input to unwrapping algorithms [11–13]. No direct comparisons with higher k_e_ have been performed to validate this approach.

Very low k_e_ may be undesirable due to low sensitivity to displacement [10, 11]. If the sensitivity is too low, there may be errors in the quantifications of cardiac mechanics. While this may be problematic as the k_e_ approaches zero, a relatively low k_e_ of 0.04 cycles/mm is still able to resolve displacements of 0.006 mm with typical 12-bit data storage. More importantly, though, the sensitivity of the displacement measurements to phase noise increases with decreasing k_e_. No study has investigated a range of k_e_ to ascertain its effects on quantifications of cardiac mechanics. It has also been suggested that a high k_e_ is required to dephase the blood pool signal [10]. This may not be the case, however, as long as a through-plane dephasing gradient is in place to accomplish the dephasing.

We hypothesized that 1) quantifications of myocardial circumferential strain, radial strain, and twist will not be different for encoding frequencies between 0.02 and 0.10 cycles/mm, 2) the nulling of the blood signal will be similar for all encoding frequencies, 3) the use of lower encoding frequencies will prevent phase wrapping even in healthy subjects with substantial cardiac motion, and 4) lower encoding frequencies will have higher SNR. We tested these hypotheses using a spiral cine DENSE protocol implemented on a 3 T Siemens Tim Trio MRI scanner.

## Methods

### Image acquisition

This protocol was approved by the local Institutional Review Board of the University of Kentucky. Ten healthy subjects (50 % female, age 27 ± 9) with no history of cardiovascular disease and ten subjects with a history of myocardial infarction or congestive heart failure (40 % female, age 57 ± 6) consented for the study. A 3 T Siemens (Erlangen, Germany) Tim Trio with a 6-element chest and 24-element spine coil was used to acquire mid-ventricular short-axis 2D cine DENSE images with the following parameters: 6 spiral interleaves, 1 average, 360 × 360 mm^2^ field of view, 128 × 128 reconstruction matrix, 2.8 × 2.8 mm^2^ pixel size, 8 mm slice thickness, 1.08 ms/17 ms TE/TR, constant 20° flip angle. Two spirals were acquired per heartbeat which yielded a temporal resolution of 34 ms. View sharing was used to achieve 17 ms between reconstructed cardiac frames. Simple encoding was used to measure in-plane displacements while through-plane dephasing of 0.08 cycles/mm and CSPAMM were used for echo suppression [3, 7, 11]. To remove effects due to variable breath-hold position, the acquisitions were performed with respiratory navigator gating and an acceptance window of ±3 mm.

In each subject, the same mid-ventricular short-axis slice was acquired five times with different values of in-plane k_e_: 0.02, 0.04, 0.06, 0.08, and 0.10 cycles/mm. The 0.10 cycles/mm acquisition was repeated during the same imaging session to assess inter-test reproducibility.

### DENSE strain and twist analyses

Myocardial strain and twist were derived from the DENSE images using custom software written in MATLAB (The Mathworks Inc, Natick, MA). The post-processing steps for each cine DENSE slice included manual segmentation of the left ventricular myocardium and semi-automated phase unwrapping to obtain the 2D Eulerian displacements within each cardiac frame [10]. Following the unwrapping, spatial smoothing and temporal fitting of displacements (10^th^ order polynomial) were performed to obtain smooth trajectories for all tissue points beginning at end-diastole and continuing through systole into much of diastole [10]. Radial strain, circumferential strain, and twist were calculated from the resulting displacement fields for each cardiac frame [14].

Radial and circumferential strains were quantified with the 2D Lagrangian finite strain tensor in six circumferential segments throughout the cardiac cycle. Radial strain was defined as positive for thickening while circumferential strain was negative for shortening. To report peak global strains, the curves from the six segments were averaged into a single global curve from which the peak was selected. Twist was quantified in the same segments and was defined as the angle of rotation about the centroid of the endocardial contour at end-diastole. Twist was positive for counterclockwise rotation when viewing the short-axis slice from the apex towards the base. Peak global twist was quantified in the same manner as the peak global strains.

As many recent studies have used a k_e_ of 0.10 cycles/mm, the peak strains and twists quantified with the other k_e_ were compared to the same measures quantified with a k_e_ of 0.10 cycles/mm. Paired t-tests (with significance defined as *p* < 0.05), Bland-Altman analyses [15], and modified coefficients of variation (CoV) were used for statistical comparison. The equation for CoV is below for a given measurement, *X*, quantified in *N* subjects with two encoding frequencies (*ke1* and *ke2*) [6, 16].

$$ CoV=\frac{{\displaystyle {\sum}_{i=1}^N}\left[ St.Dev.{\left({X}_{ke1\ }\ {X}_{ke2}\right)}_i\right]/N}{\left|{\displaystyle {\sum}_{i=1}^N}\left[{\left(\left({X}_{ke1}+{X}_{ke2}\right)/2\right)}_i\right]/N\right|} $$

### Phase wrapping

The amount of phase wrapping that occurred for a given subject and k_e_ was measured by first considering the phase images for the X and Y directions separately. For each of the two directions, the cardiac frame with the largest percentage of wrapped pixels within the cardiac segmentation was found. The cardiac frame with this largest percentage may have been at slightly different time points for the two directions, though always near end-systole because that is when the most displacement and wrapping occurred. The average of those two percentages was taken as the amount of phase wrapping for that subject and k_e_.

### Blood pool dephasing

Dephasing of the blood signal through the cardiac cycle for each k_e_ was quantified by calculating the average pixel intensity of the DENSE magnitude images within a set of manually defined contours that denoted the blood pool. Care was taken to ensure that the papillary muscles and trabeculations were not included within the blood pool for this analysis. The magnitude of the blood pool signal was quantified and expressed through the cardiac cycle as a percentage of its signal in the first cardiac phase. To demonstrate the amount of dephasing that has occurred by early systole, the blood pool signal remaining at the fifth cardiac frame (85 ms into the cardiac cycle) was compared between the acquisitions with different k_e_

### Signal to noise ratio

To compare the effects of intra-voxel dephasing between the different k_e_, the signal to noise ratio (SNR) was calculated for each cardiac phase. The end-systolic SNR for each lower k_e_ was compared to the SNR for k_e_ of 0.10 cycles/mm with a paired *t*-test. SNR was calculated from the magnitude images by finding the average signal within the myocardium and the standard deviation (noise) of signal within a region of zero signal outside of the body. Care was taken to avoid image artifacts in the region of zero signal. Corrections were applied for the Rician distribution of the MR signal [17]. The true standard deviation of the signal, σ, was calculated from the measured standard deviation, σ_M_, by

$$ \sigma = \sqrt{\frac{2}{4-\pi }}*{\sigma}_M \approx 1.526*{\sigma}_M $$

The true myocardial signal, *S*, was calculated from the measured myocardial signal, M, by

$$ S = \sqrt{M^2-{\sigma}^2} $$

SNR was calculated as the ratio of *S* to σ.

### Relationship between phase noise and SNR

To assess the relationship between phase noise and SNR, the same DENSE acquisitions above were performed on a stationary water phantom. SNR was quantified in the same manner as for the human studies. For each k_e_, the phase noise in the X and Y phase images was quantified via the root mean squared error (RMSE) in radians. To compute the RMSE of the 2D displacements, the previous RMSEs were converted from radians to millimeters via the k_e_. The X and Y RMSEs in millimeters were then added together via vector addition to yield the 2D RMSE. The phase noise in radians is theoretically inversely proportional to the SNR [17].

## Results

As quantified by the DENSE acquisition with a k_e_ of 0.10 cycles/mm, the patients had a mean (± standard deviation) global circumferential strain of −12 ± 6 % (range: −3 to −20 %). The same measure in the healthy subjects was −20 ± 2 % (range: −17 to −23 %).

**Fig. 2:**
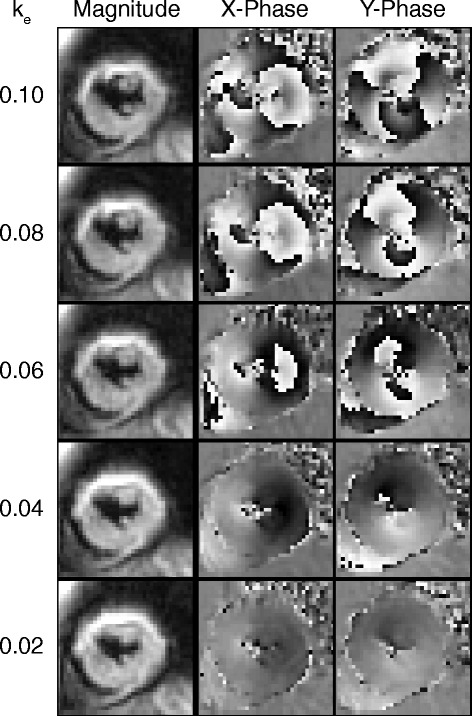
End-systolic magnitude and phase images from a subject with previous myocardial infarction. Substantial wrapping was present in the phase images for the higher k_e_. As the k_e_ was decreased, the amount of wrapping in the X and Y phase images decreased. No wrapping was present in the myocardium for 0.02 and 0.04 cycles/mm. Also note that the blood pool dephased similarly for all k_e_

End-systolic images from a representative subject are shown in Fig. 2 and demonstrate a reduction in phase wrapping at lower k_e_. No phase wrapping was present within the segmentation of the myocardium for k_e_ of 0.04 and 0.02 cycles/mm.

### DENSE strain and twist analyses

**Fig. 3:**
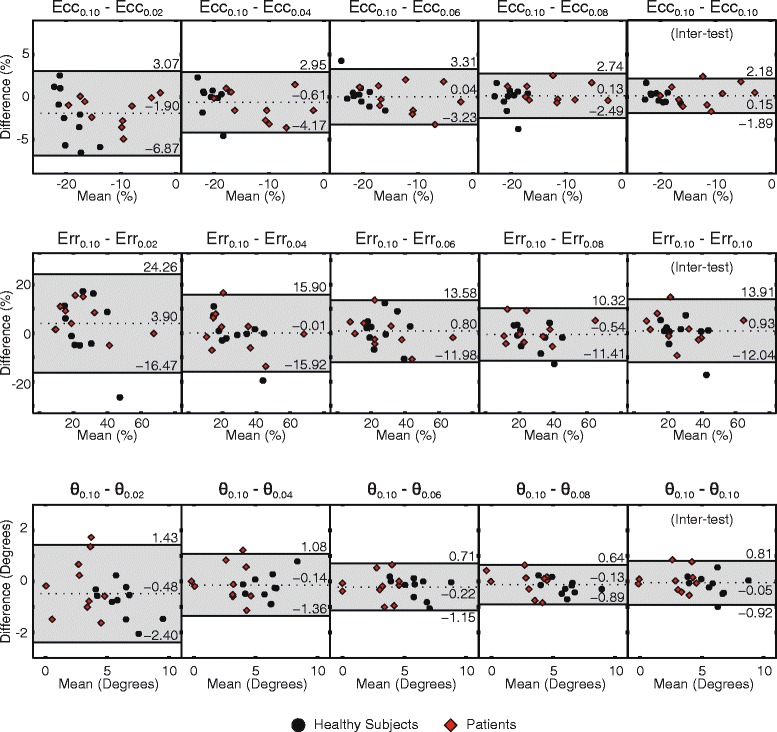
Bland-Altman plots demonstrate agreement among k_e_ of at least 0.04 cycles/mm. The first, second, and third rows contain Bland-Altman plots for circumferential strain (Ecc), radial strain (Err), and twist (θ), respectively. The subscript values denote the comparisons between acquisitions with the stated k_e_. The inter-test comparison was between two acquisitions with k_e_ of 0.10 cycles/mm. The shaded areas denote the region within the 95 % limits of agreement. The worst agreement was seen between 0.02 and 0.10 cycles/mm

Negligible differences were seen in strains and twist for all k_e_ between 0.04 and 0.10 cycles/mm (Fig. 3, Table 1). These differences were of the same magnitude as inter-test differences. The comparison between k_e_ of 0.02 and 0.10 cycles/mm, however, demonstrated larger biases, larger 95 % limits of agreement (LoA), and larger CoVs for both strains and twist. The differences in circumferential strain and twist between k_e_ of 0.02 and 0.10 cycles/mm were significant (*p* = <0.01 and *p* = 0.04, respectively).

**Table 1:** Summary statistics showed good agreement for all k_e_ between 0.04 and 0.10 cycles/mm. Larger biases, 95 % LoA, and CoVs were observed for k_e_ of 0.02 cycles/mm

E(k_e_)**	Circumferential Strain (%)				Radial Strain (%)				Twist (Degrees)			
	Bias	95 % LoA	Mean CoV	*p*-value	Bias	95 % LoA	Mean CoV	*p*-value	Bias	95 % LoA	Mean CoV	*p*-value
E_0.10_ – E_0.02_	−1.9	±5.0	11 %	<0.01*	3.9	±20.4	23 %	0.11	−0.48	±1.92	14 %	0.04*
E_0.10_ – E_0.04_	−0.6	±3.6	6 %	0.15	−0.0	±15.9	14 %	1.00	−0.14	±1.22	8 %	0.32
E_0.10_ – E_0.06_	0.0	±3.2	6 %	0.91	0.8	±12.8	13 %	0.59	−0.22	±0.93	6 %	0.05
E_0.10_ – E_0.08_	0.1	±2.6	4 %	0.67	−0.5	±10.9	11 %	0.67	−0.13	±0.77	5 %	0.16
Inter-test	0.1	±2.0	4 %	0.53	0.9	±13.0	12 %	0.54	−0.05	±0.87	5 %	0.59

*Statistical significance between peak measures of mechanics using paired-sample *t*-test at significance level α = 0.05

**${E}_{\left[{\boldsymbol{k}}_{\boldsymbol{e}}\right]}$ represents peak strain or twist measured using a particular k_e_

Abbreviations: LoA *limits of agreemen*t, CoV *coefficient of variation*

### Phase wrapping

For k_e_ of 0.02, 0.04, 0.06, 0.08, and 0.10 cycles/mm, the largest percentage of wrapped pixels in the phase images was 0 ± 0, 0 ± 0, 5 ± 6, 17 ± 10, and 32 ± 9 %, respectively. Thus, phase images acquired with a k_e_ of 0.04 cycles/mm had zero wrapped pixels. In contrast, the same phase images acquired with a k_e_ of 0.10 cycles/mm had about 32 % of the pixels wrapped in the cardiac frame with the most displacement.

### Blood pool dephasing

**Fig. 4:**
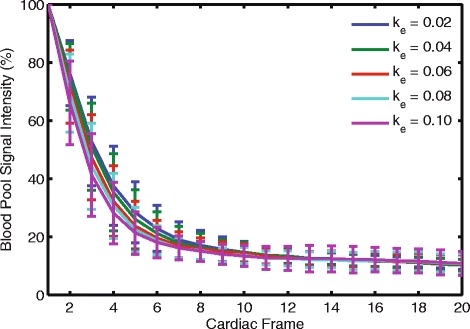
Similar rates of blood pool dephasing were observed for the different k_e_. Blood pool signal intensity was expressed as a percentage of its value at the first cardiac phase. The first 20 cardiac frames are shown. Each curve represents the average of the 20 subjects with standard deviation error bars. As the k_e_ increased, the rate of blood pool dephasing increased, but with considerable overlap between the different k_e_ as seen by the wide standard deviation bars

As k_e_ increased, the rate of blood pool dephasing increased, however, the standard deviations demonstrated considerable overlap among the different k_e_ (Fig. 4). Across the 20 subjects and using the fifth cardiac phase as an example, the amount of blood pool signal remaining as a percentage of its initial value was 28 ± 11, 26 ± 10, 24 ± 9, 23 ± 8, and 21 ± 7 % for k_e_ of 0.02, 0.04, 0.06, 0.08, and 0.10 cycles/mm, respectively. Frame 20 was the average end-systolic frame and there was no effective difference in blood pool dephasing by that time.

### Signal to noise ratio

**Fig. 5:**
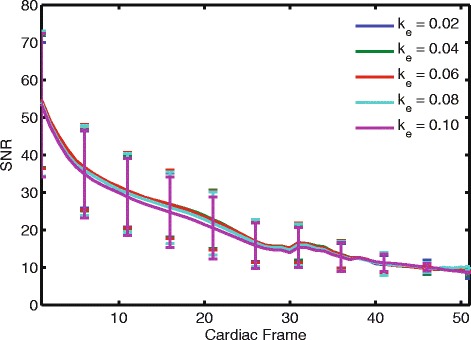
The SNR throughout the cardiac cycle was similar for the different k_e_. Each curve represents the average of the 20 subjects with standard deviation error bars. Starting with the first frame, the standard deviation is shown at every fifth cardiac frame for clarity. There is a trend towards higher SNR at lower k_e_, particularly between the 15^th^ and 20^th^ cardiac frames

SNR throughout the cardiac cycle was similar for the different k_e_ (Fig. 5), with a trend towards higher SNR at lower k_e_. Across the 20 subjects, the mean SNR at end-systole, which occurred at different cardiac frames for the different subjects, was 23 ± 9, 24 ± 9, 23 ± 9, 23 ± 10, and 22 ± 9 for k_e_ of 0.02, 0.04, 0.06, 0.08, and 0.10 cycles/mm, respectively. The end-systolic SNR for k_e_ = 0.02, 0.04, 0.06, and 0.08 were each significantly different than the end-systolic SNR for k_e_ = 0.10 cycles/mm (*p* = 0.010, 0.003, 0.005, 0.03, respectively). This represents a 9 % increase in SNR for k_e_ of 0.04 cycles/mm compared to a k_e_ of 0.10 cycles/mm.

### Relationship between phase noise and SNR

**Fig. 6:**
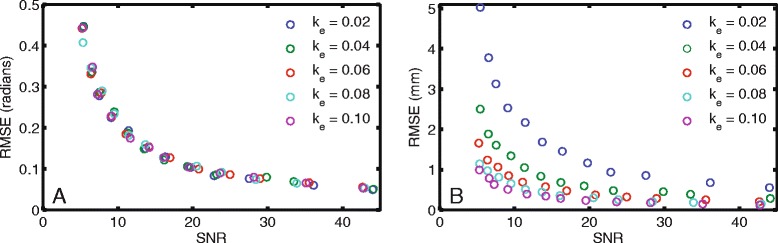
Phase noise had a larger effect on displacement errors with lower k_e_. **a** In a stationary water phantom, phase noise, as quantified by RMSE in radians, was inversely related to SNR. No differences in RMSE were seen between the different k_e_. **b** When RMSE in radians was converted to millimeters by dividing by k_e_, there were substantial differences between the different k_e_. Lower k_e_ had increased displacement errors

In the stationary water phantom, the inverse relationship between the phase noise (as measured by RMSE in radians) and the SNR was similar for all k_e_ (Fig. 6a). However, the RMSE in millimeters, which required division by the appropriate k_e_, was substantially higher for lower k_e_ (Fig. 6b). For example, for SNR near 20, the RMSEs in millimeters were 1.17, 0.60, 0.38, 0.30, and 0.23 mm, for k_e_ = 0.02, 0.04, 0.06, 0.08, and 0.10 cycles/mm, respectively.

## Discussion

Spiral 2D cine DENSE has typically been acquired with a k_e_ of 0.10 cycles/mm [5, 8, 9]. This value is high enough to cause phase wrapping after only 5 mm of tissue displacement. In the present study, we investigated the hypothesis that lower k_e_ could be used to reduce the amount of phase wrapping without compromising the quantification of strain and twist from mid-ventricular short-axis images. Our primary findings included: 1) the k_e_ can be reduced to 0.04 cycles/mm without causing differences in the quantifications of circumferential strain, radial strain, or twist; 2) phase wrapping can be eliminated from the phase images with the use of k_e_ less than or equal to 0.04 cycles/mm; 3) the rate of blood pool dephasing, which is a source of contrast between blood and myocardium in the magnitude images, is similar for k_e_ between 0.02 and 0.10 cycles/mm; and 4) the SNR at end-systole is 9 % higher when using a k_e_ of 0.04 cycles/mm compared to using a k_e_ of 0.10 cycles/mm.

### DENSE strain and twist analyses

Spiral cine DENSE is primarily used to measure cardiac displacements and deformation in the forms of twist and strain [3, 10, 12]. The k_e_ is the proportionality constant between the tissue displacement in millimeters and the measured signal phase. It also determines the strength of the encoding gradient that is applied. A high value of k_e_ provides high sensitivity to small displacements, but at the cost of intra-voxel dephasing and increased phase wrapping. The results from this study suggest that the k_e_ can be lowered to 0.04 cycles/mm, which significantly reduces the presence of phase wrapping, without compromising measures of circumferential strain, radial strain, or twist. In addition, studies that use different k_e_ between 0.04 and 0.10 cycles/mm can be directly compared as no systematic differences in strain or twist due to differences in k_e_ were found. This is valuable as not all DENSE studies have used the typical value of 0.10 cycles/mm. In particular, some previous studies have used 0.06 cycles/mm [12, 13], which is within the range of this study.

The measures of strain and twist were compromised as the k_e_ was lowered to 0.02 cycles/mm (Fig. 3). This was likely caused by the increased effect of phase errors at low k_e_ (Fig. 6b). For a given phase error in radians, the corresponding error in displacement (mm) was larger for lower k_e_. This same phenomenon is present in phase contrast velocity imaging as the velocity encoding (VENC) is increased [18].

### Phase wrapping

The amount of phase wrapping decreased as the k_e_ was decreased. Lowering the k_e_ to the point that there is no wrapping puts DENSE on a similar level as phase contrast velocity imaging, where the VENC is commonly adjusted to prevent wrapping in the blood velocities [19]. The use of this low value was possible due to the artifact suppression techniques of CSPAMM [3] and through-plane dephasing [7]. As seen in the representative subject (Fig. 2), no stripe artifacts were present in the images for the low k_e_.

### Blood pool dephasing

The rate of blood pool dephasing decreased as the k_e_ was decreased (Fig. 4). However, the difference between the acquisitions with 0.10 and 0.02 cycles/mm was not large. By the fifth cardiac frame, the acquisition with 0.02 cycles/mm had approximately 7 % more of its blood pool signal remaining. This difference was not practically significant as the delineation between the myocardium and the blood pool was still possible at the lowest k_e_. The drop in blood pool signal through the cardiac cycle is due to dephasing [10]. This dephasing can be due to both in-plane and through-plane gradients. While the in-plane gradients necessarily changed with the k_e_, the through-plane gradient remained constant for all acquisitions and likely contributed to the blood pool dephasing at similar rates for all k_e_. Thus, the advent of through-plane dephasing removed dependence on high k_e_ to accomplish blood pool dephasing.

### Signal to noise ratio

The SNR was 9 % higher for k_e_ of 0.04 cycles/mm compared to 0.10 cycles/mm. This is a reflection of the decreased intra-voxel dephasing that occurs due to the decreased gradient strengths that accompany lower k_e_. This modest increase in SNR is generally beneficial and results in a reduction in phase noise [17].

It is important to note that both the in-plane encoding gradient and the through-plane dephasing gradient are capable of producing intra-voxel dephasing of the stimulated echo in deforming tissue [7]. The voxel size in the through-plane direction was larger than the in-plane direction (8 mm vs. 2.8 mm). Thus, the amount of intra-voxel dephasing may have been largely controlled by the through-plane dephasing gradient, which was constant (0.08 cycles/mm) for all acquisitions in this study. Further increases in SNR could be possible by reducing the through-plane dephasing gradient, however, this value was chosen to cause more than one half cycle of dephasing across the 8 mm slice [7]. Reducing the amount of through-plane dephasing could lead to the presence of stripe artifacts in the images.

### Limitations

This study assessed a single mid-ventricular short-axis slice without consideration of long-axis images. The longitudinal motion of the left ventricle (particularly near the base) is often larger than the circumferential and radial components [20]. Long-axis images would likely have demonstrated phase wrapping with a k_e_ of 0.04 cycles/mm. While this implies that unwrapping algorithms cannot be removed from the post-processing, the amount of wrapping can be substantially reduced with a lower value. As the circumferential and radial strains were not compromised in the short-axis images with this low value, the longitudinal strains from the long-axis images should also not be compromised.

The acquisitions in this study were performed at 3 T, which yields higher SNR compared to 1.5 T [21]. Acquisitions at 1.5 T may have larger phase errors (due to decreased SNR) than those present in this study. However, those errors could be offset by better field homogeneity at the lower field strength. It has recently been reported that the displacement errors from spiral cine DENSE are the same at 3 T and 1.5 T [16]. Thus, the results from this study are likely applicable to 1.5 T.

We performed the acquisitions in this study with the simple encoding strategy because of the reported ability to handle phase wrapping due to k_e_ as high as 0.10 cycles/mm [10]. A motivation for this study, however, was to investigate the ability to lower the k_e_ during acquisitions that use the balanced encoding strategy. This strategy has been used for DENSE acquisitions that encode displacements in all three directions [11–13]. However, in those studies, the k_e_ was reduced to 0.06 cycles/mm due to the increased wrapping that is present in the online reconstructed images [11]. We could not guarantee successful unwrapping from images acquired with the balanced strategy and a k_e_ of 0.10 cycles/mm, so the simple strategy was used to be able to accurately test up to 0.10 cycles/mm. The results from this study suggest that the k_e_ could likely be lowered to 0.04 cycles/mm with the balanced strategy, which has better noise performance than the simple encoding strategy [11]. This lower value would reduce the load on the unwrapping algorithm for 3D DENSE studies and any DENSE studies that use the balanced encoding strategy. The strain and twist results from this study suggest that these measures of cardiac mechanics would not be compromised with the lower value.

## Conclusions

Cine DENSE is typically acquired with an encoding frequency of 0.10 cycles/mm [5, 8, 9]. This value allows for high sensitivity to tissue displacements, but at the cost of substantial phase wrapping. We demonstrated that the encoding frequency can be lowered to 0.04 cycles/mm to nearly eliminate phase wrapping without compromising the quantification of cardiac strains or twist. Future studies may take advantage of this lower value to reduce the amount of wrapping and simplify the input to unwrapping algorithms. In addition, studies performed with different encoding frequencies between 0.04 and 0.10 cycles/mm can be directly compared as there is no systematic bias.

## Abbreviations

CMR: Cardiovascular magnetic resonance
CoV: Coefficient of variation
CSPAMM: Complementary spatial modulation of magnetization
DENSE: Displacement encoding with stimulated echoes
k_e_: Encoding frequency
MR: Magnetic resonance
RMSE: Root mean squared error
SNR: Signal to noise ratio
TE: Echo time
TR: Repetition time
VENC: Velocity encoding

## References

[1] Aletras AH, Ding S, Balaban RS, Wen H (1999). DENSE: displacement encoding with stimulated echoes in cardiac functional MRI. J Magn Reson.

[2] Aletras AH, Balaban RS, Wen H (1999). High-resolution strain analysis of the human heart with fast-DENSE. J Magn Reson.

[3] Kim D, Gilson WD, Kramer CM, Epstein FH (2004). Myocardial tissue tracking with two-dimensional cine displacement-encoded MR imaging: development and initial evaluation. Radiology.

[4] Ernande L, Thibault H, Bergerot C, Moulin P, Wen H, Derumeaux G, Croisille P (2012). Systolic myocardial dysfunction in patients with type 2 diabetes mellitus: identification at MR imaging with cine displacement encoding with stimulated echoes. Radiology.

[5] Bilchick KC, Kuruvilla S, Hamirani YS, Ramachandran R, Clarke SA, Parker KM, Stukenborg GJ, Mason P, Ferguson JD, Moorman JR, Malhotra R, Mangrum JM, Darby AE, Dimarco J, Holmes JW, Salerno M, Kramer CM, Epstein FH (2014). Impact of mechanical activation, scar, and electrical timing on cardiac resynchronization therapy response and clinical outcomes. J Am Coll Cardiol.

[6] Haggerty CM, Kramer SP, Binkley CM, Powell DK, Mattingly AC, Charnigo R, Epstein FH, Fornwalt BK (2013). Reproducibility of cine displacement encoding with stimulated echoes (DENSE) cardiovascular magnetic resonance for measuring left ventricular strains, torsion, and synchrony in mice. J Cardiovasc Magn Reson.

[7] Zhong X, Spottiswoode BS, Cowart EA, Gilson WD, Epstein FH (2006). Selective suppression of artifact-generating echoes in cine DENSE using through-plane dephasing. Magn Reson Med.

[8] Young AA, Li B, Kirton RS, Cowan BR (2012). Generalized spatiotemporal myocardial strain analysis for DENSE and SPAMM imaging. Magn Reson Med.

[9] Budge LP, Helms AS, Salerno M, Kramer CM, Epstein FH, Bilchick KC (2012). MR cine DENSE dyssynchrony parameters for the evaluation of heart failure: comparison with myocardial tissue tagging. JACC Cardiovasc Imaging.

[10] Spottiswoode BS, Zhong X, Hess AT, Kramer CM, Meintjes EM, Mayosi BM, Epstein FH (2007). Tracking myocardial motion from cine DENSE images using spatiotemporal phase unwrapping and temporal fitting. IEEE Trans Med Imaging.

[11] Zhong X, Helm PA, Epstein FH (2009). Balanced multipoint displacement encoding for DENSE MRI. Magn Reson Med.

[12] Zhong X, Spottiswoode BS, Meyer CH, Kramer CM, Epstein FH (2010). Imaging three-dimensional myocardial mechanics using navigator-gated volumetric spiral cine DENSE MRI. Magn Reson Med.

[13] Auger DA, Zhong X, Epstein FH, Spottiswoode BS (2012). Mapping right ventricular myocardial mechanics using 3D cine DENSE cardiovascular magnetic resonance. J Cardiovasc Magn Reson.

[14] Zhong X, Gibberman LB, Spottiswoode BS, Gilliam AD, Meyer CH, French BA, Epstein FH (2011). Comprehensive cardiovascular magnetic resonance of myocardial mechanics in mice using three-dimensional cine DENSE. J Cardiovasc Magn Reson.

[15] Bland JM, Altman DG (1986). Statistical methods for assessing agreement between two methods of clinical measurement. Lancet.

[16] Wehner GJ, Suever JD, Haggerty CM, Jing L, Powell DK, Hamlet SM, Grabau JD, Mojsejenko WD, Zhong X, Epstein FH, Fornwalt BK (2015). Validation of in vivo 2D displacements from spiral cine DENSE at 3T. J Cardiovasc Magn Reson.

[17] Gudbjartsson H, Patz S (1995). The Rician distribution of noisy MRI data. Magn Reson Med.

[18] Pelc NJ, Sommer FG, Li KC, Brosnan TJ, Herfkens RJ, Enzmann DR (1994). Quantitative magnetic resonance flow imaging. Magn Reson Q.

[19] Nett EJ, Johnson KM, Frydrychowicz A, Del Rio AM, Schrauben E, Francois CJ, Wieben O (2012). Four-dimensional phase contrast MRI with accelerated dual velocity encoding. J Magn Reson Imaging.

[20] Moore CC, Lugo-Olivieri CH, McVeigh ER, Zerhouni EA (2000). Three-dimensional systolic strain patterns in the normal human left ventricle: characterization with tagged MR imaging. Radiology.

[21] Sigfridsson A, Haraldsson H, Ebbers T, Knutsson H, Sakuma H (2011). In vivo SNR in DENSE MRI; temporal and regional effects of field strength, receiver coil sensitivity and flip angle strategies. Magn Reson Imaging.

